# PSMD1 as a prognostic marker and potential target in oropharyngeal cancer

**DOI:** 10.1186/s12885-023-11689-2

**Published:** 2023-12-16

**Authors:** Hae Chan Park, Hyojin Kim, Ji-Yeong Kim, Hye-Yeon Lee, Jinyi Lee, WonJae Cha, Soon-Hyun Ahn, Woo-Jin Jeong

**Affiliations:** 1 Department of Otorhinolaryngology-Head & Neck Surgery, Seoul National University Bundang Hospital, Seoul National University College of Medicine, Seongnam, Korea; 2 Department of Pathology, Seoul National University Bundang Hospital, Seoul National University College of Medicine, Seongnam, Korea; 3 Department of Otorhinolaryngology-Head and Neck Surgery, Seoul National University College of Medicine, Seoul, Korea; 4 Sensory Organ Research Institute, Seoul National University Medical Research Center, Seoul, Korea

**Keywords:** Head and neck cancer, Proteasome inhibitor, Targeted therapy, Immunohistochemistry, HPV, Oropharynx cancer

## Abstract

**Background:**

Despite the diverse genetic mutations in head and neck cancer, the chemotherapy outcome for this cancer has not improved for decades. It is urgent to select prognostic factors and therapeutic targets for oropharyngeal cancer to establish precision medicine. Recent studies have identified PSMD1 as a potential prognostic marker in several cancers. We aimed to assess the prognostic significance of PSMD1 expression in oropharyngeal squamous cell carcinoma (OPSCC) patients using immunohistochemistry.

**Methods:**

We studied 64 individuals with OPSCC tissue from surgery at Seoul National University Bundang Hospital between April 2008 and August 2017. Immunostaining analysis was conducted on the tissue microarray (TMA) sections (4 μm) for p16 and PSMD1. H-score, which scale from 0 to 300, was calculated from each nucleus, cytoplasm, and cellular expression. Clinicopathological data were compared with Chi-squared test, Fisher’s exact test, t-test, and logistic regression. Survival data until 2021 were achieved from national statistical office of Korea. Kaplan–Meier method and cox-regression model were used for disease-specific survival (DSS) analysis.

**Results:**

H-score of 90 in nucleus was appropriate cutoff value for ‘High PSMD1 expression’ in OPSCC. Tonsil was more frequent location in low PSMD1 group (42/52, 80.8%) than in high PSMD1 group (4/12, 33.3%; *P* = .002). Early-stage tumor was more frequent in in low PSMD1 group (45/52, 86.5%) than in high PSMD1 group (6/12, 50%; *P* = .005). HPV was more positive in low PSMD1 group (43/52, 82.7%) than in high PSMD1 group (5/12, 41.7%; *P* = .016). Patients with PSMD1 high expression showed poorer DSS than in patients with PSMD1 low expression (*P* = .006 in log rank test). In multivariate analysis, PSMD1 expression, pathologic T staging, and specimen age were found to be associated with DSS (*P* = .011, *P* = .025, *P* = .029, respectively).

**Conclusions:**

In our study, we established PSMD1 as a negative prognostic factor in oropharyngeal squamous cell carcinoma, indicating its potential as a target for targeted therapy and paving the way for future in vitro studies on drug repositioning.

**Supplementary Information:**

The online version contains supplementary material available at 10.1186/s12885-023-11689-2.

## Introduction

Despite the diverse genetic mutations and the presence of HPV in head and neck cancer, the chemotherapy for this cancer has traditionally been based on cisplatin, and treatment outcomes have not improved for decades [1, 2]. Cisplatin-based chemotherapy is non-specific and has significant side effects. Therefore, the development of targeted therapeutic agents with fewer side effects and related biomarkers is necessary.

Proteasomes are crucial intracellular proteins that regulate the degradation of ubiquitinated proteins [3], and their complex composition of highly regulated proteins makes them attractive candidate for new targeted cancer therapy [4]. PSMD1 is a suitable research target for drug repositioning since there is already an FDA-approved drug called proteasome inhibitor, which is mostly used to treat refractory cases of multiple myeloma [5]. Indeed, researchers are currently investigating the potential of proteasome inhibitors like Bortezomib and Carfilzomib to be utilized in the treatment of different types of solid malignancies [6]. Recent studies also have identified PSMD1, which encodes a subunit of the proteasome, as an upregulated factor and potential prognostic marker in several cancers including anaplastic thyroid carcinoma [7], breast cancer [8], gastric cancer [9], colon cancer [10], ovarian cancer [11], among others [12]. However, the significance of PSMD1 expression in head and neck cancer remains to be elucidated.

Therefore, the purpose of this study is to assess the prognostic significance of PSMD1 expression in oropharyngeal squamous cell carcinoma (OPSCC) patients using immunohistochemistry. Using clinicopathological data, we aimed to determine the clinical value of PSMD1 expression. Additionally, we investigated disease-specific survival (DSS) of OPSCC patients, considering both PSMD1 expression and clinicopathological information.

## Materials and methods

### Patients and tissue specimens

We included 64 individuals with OPSCC tissue confirmed by curative resection or diagnostic surgical biopsy at Seoul National University Bundang Hospital between April 2008 and August 2017. We excluded patients who were currently receiving or had previously received treatment for other types of squamous cell carcinoma (SCC) in the head and neck area, as well as those with histological findings that differed from SCC or its subtypes.

The clinicopathological data of the patients were obtained, including age, gender, tobacco and alcohol use, tumor subsite, surgical intent, tumor recurrence, postoperative treatment, and status at the last follow-up. Pathologic stages were classified based on the 8th edition AJCC staging system [13]. The research protocol received approval from the Institutional Review Board of Seoul National University Bundang Hospital, and informed consent was waived (IRB No. B-2211–790-305).

Tissue microarrays (TMAs) were constructed from formalin-fixed paraffin-embedded blocks of specimens. Core tissue sections (with a diameter of 4 mm) were carefully extracted from individual OPSCC paraffin blocks (donor blocks) and organized in new TMA blocks using a trephine apparatus (Super-BioChips Laboratories, Seoul, Korea). To reduce the impact of heterogeneity of protein expression, three cores were sampled and incorporated into the TMA block from each patient.

### HPV genotyping

HPV status was determined by HPV genotyping using the complete resected section and biopsy specimens. HPV genotyping was performed using peptic nucleic acid probe-based fluorescence melting curve analysis in a real-time PCR system (PANA RealTyper™ HPV Kit, PANAGENE, Daejeon, Republic of Korea) according to the manufacturer’s instructions as described in previous study [14].

### Assessment of PSMD1 protein expression

Immunohistochemistry for PMSD1 IHC analysis was conducted on the TMA sections (4 μm) using an automated platform (Benchmark Ultra; Ventana Medical Systems) following the manufacturer's instructions. For PSMD1 immunostaining, a rabbit polyclonal IgG specific to Human PSMD1 (Abcam, ab2941, 1:5000) was employed. At a random × 40 magnification field within the tumor, the nucleus, cytoplasm, and cellular DAB staining intensity in the malignant cells were quantified using an image analyzer (QuPath) for H-score interpretation. If more than 10% of the tumor cells were 1 + , 2 + , or 3 + , the tumor was graded as 1 + , 2 + , or 3 + , respectively. If less than 10% of tumor was 1 + or higher, the tumor was graded as 0 (negative). The total number of cells stained at each grade within the field was counted. Subsequently, the following formula was applied: H-score = (% of 1 + cells × 1) + (% of 2 + cells × 2) + (% of 3 + cells × 3). Consequently, an H-score ranging from 0 to 300 was obtained, where 300 represented 100% of tumor cells exhibiting strong staining (3 +).

### Statistical analysis

All data analysis was conducted using SPSS version 25.0 (SPSS Inc., Chicago, IL, USA). The determination of appropriate cut-off values for the H-score was performed using receiver operating characteristic (ROC) curve analysis. Chi-squared test, Fisher’s exact test, t-test, and logistic regression were performed to compare clinicopathological data among groups. Pilot survival data were captured from The Cancer Genome Atlas (TCGA) [15]. Survival data until 2021 were achieved from national statistical office. Kaplan–Meier method and the log-rank test were used to estimate disease-specific survival (DSS). Multivariate analysis was conducted using the Cox proportional hazards regression model. A *p*-value less than 0.05 was considered statistically significant.

## Results

### Overexpression of PSMD1 in human OPSCC

**Fig. 1:**
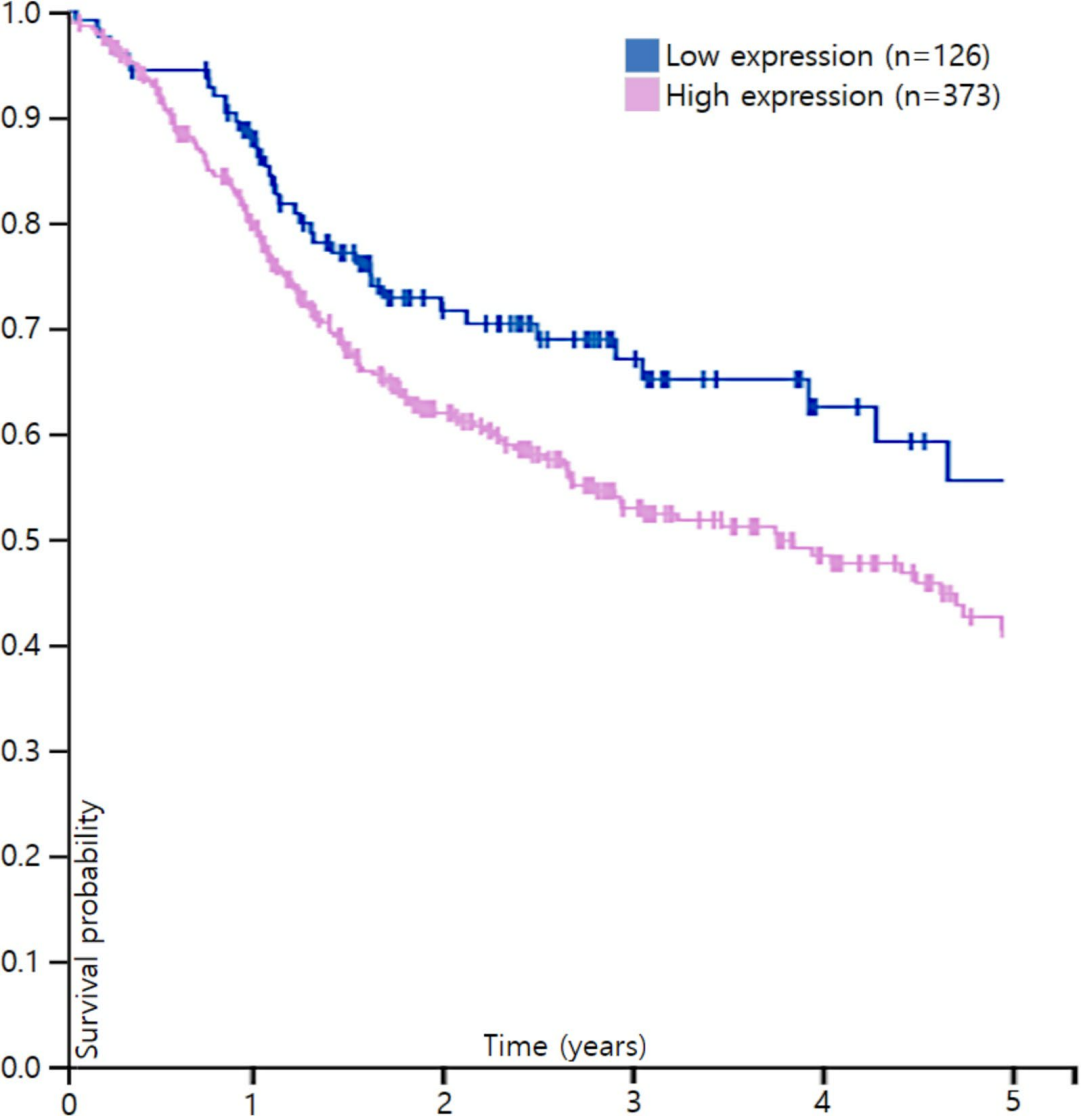
Survival analysis with respect to PSMD1 expression in head and neck cancer patient from TCGA data. Y axis stands for overall survival (OS) probability

**Fig. 2:**
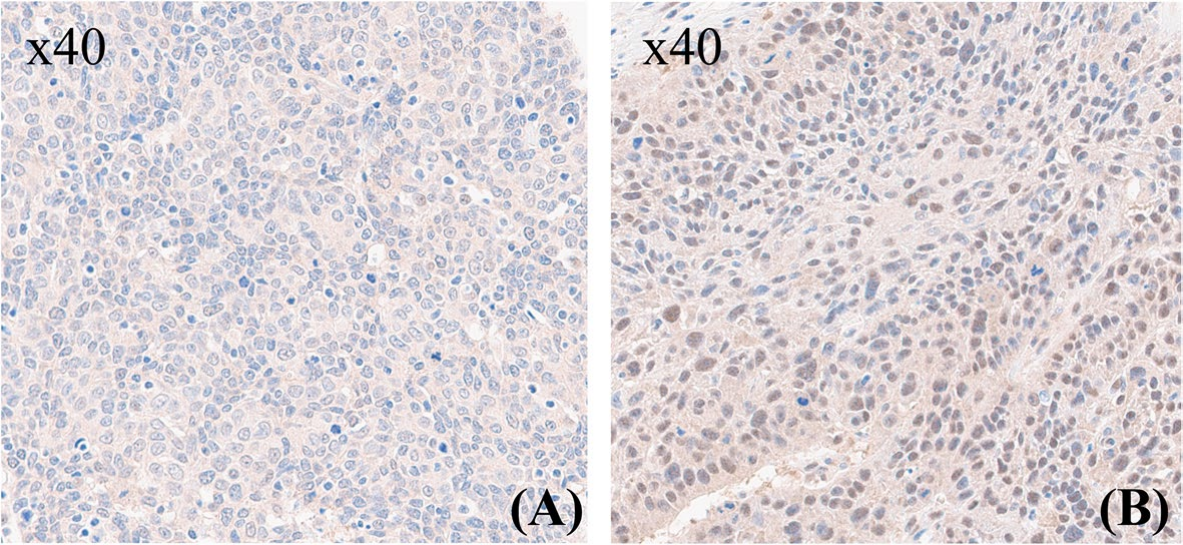
PSMD1 expression in OPSCC tissue. Pathologic images (× 40 magnification) of (**A**) Low PSMD1 expression in HPV positive OPSCC tissue compared to (**B**) high PSMD1 expression in HPV negative OPSCC tissue

For a pilot evaluation, we first comprehensively analyzed data from The Cancer Genome Atlas (TCGA, Fig. 1, [15]). In Kaplan–Meier survival curves of head and neck cancer, separation of median overall survival and log rank test *p*-value were best fitted in expression of 23.12 (FPKM). The curves didn’t cross in 5-year overall survival. PSMD1 High expression group showed lower overall survival (OS) with statistical significance in log-rank test with *p*-value of 0.018, suggesting PSMD1 as poor prognostic factor. Representative pathologic images from our specimens are illustrated in Fig. 2. Certain specimens evidently showed high PSMD1 expression.

### Determining cutoff value and target staining localization

In order to establish a new cutoff for 'high expression' of PSMD1 in two head and neck cancers with limited previous studies, we plotted ROC curves using the H-scores from each location, based on the 5-year overall survival of the patients. Area under curve (AUC) was 0.656 in nuclear H-score, 0.470 in cytoplasmic H-score, and 0.526 in cellular H-score, respectively (Supplementary Fig. 1). Consequently, between the three cellular location, nuclear H-score was the best parameter that expects survival outcome. The H-score of 90 in nucleus had the highest (sensitivity + specificity) value. Therefore H-score of 90 in nucleus was selected as a cutoff value for ‘High expression’ in further statistical analysis.

### PSMD1 expression and clinicopathologic features

The clinicopathological features of the patients are summarized in Supplementary Table 1. Briefly, the majority of the patients were men (59/64, 92.2%), and more than half had history of smoking (35/64, 54.7%) and/or alcohol (38/64, 59.4%). Of the 64 patients, 53 (82.8%) underwent curative surgery, while other 11 (17.2%) underwent limited surgery for such as diagnostic biopsy.

**Fig. 3:**
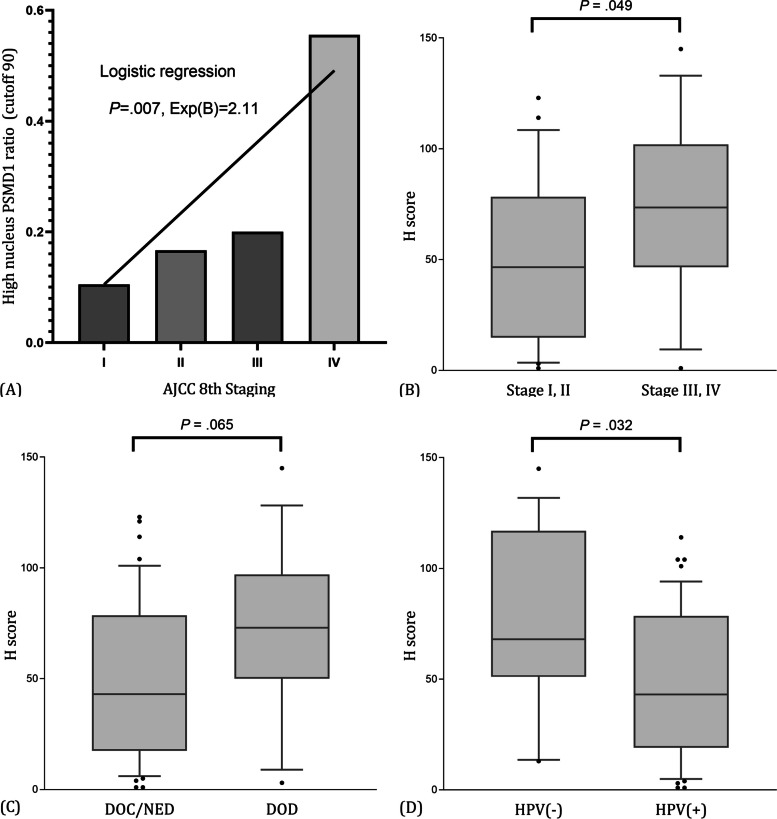
PSMD1 nuclear expression was related to advanced staging and poor survival outcome. **A** The ratio of nucleus PSMD1 'high expression', the cutoff of which was determined as 90 in ROC curve analysis (supplementary fig. 1), in each overall staging. **B** Comparison of nuclear PSMD1 expression between early stage and late-stage group. **C** Comparison of nuclear PSMD1 expression by last follow-up status. **D** Comparison of nuclear PSMD1 expression by HPV positivity. (Displaying the 90/10 percentile at the whiskers, the 75/25 percentiles at the boxes, and the median in the center line)

As the pathologic staging advance, the ratio of PSMD1 'high expression' linearly increased. High expression rate was 0.11 in stage I, 0.17 in stage II, 0.25 in stage III, and 0.56 in stage IV. Linear relationship was statistically significant in logistic regression (*P* = .007) (Fig. 3A). Nuclear H score was higher in advanced stage (48.75 ± 34.65) compared to early-stage group (75.54 ± 41.95). This difference was statistically significant (*P* = .049) (Fig. 3B). Nuclear H score was relatively higher in died of disease (DOD) group (48.96 ± 35.58) compared to death from other causes (DOC) and no evidence of disease (NED) group (71.27 ± 39.74). However, this difference was statistically insignificant (*P* = .065) (Fig. 3C). Nuclear H score was higher in HPV negative group (74.87 ± 41.56) compared to HPV positive group (47.86 ± 34.16). This difference was statistically significant (*P* = .032) (Fig. 3D).

With previously determined nuclear H-score cutoff value of 90, dichotomized expression degree and clinicopathological variables were compared in 2 × 2 table. Tonsil was more frequent location in low PSMD1 group (42/52, 80.8%) than in high PSMD1 group (4/12, 33.3%; *P* = .002). Early-stage tumor was more frequent in in low PSMD1 group (45/52, 86.5%) than in high PSMD1 group (6/12, 50%; *P* = .005). HPV was more positive in low PSMD1 group (43/52, 82.7%) than in high PSMD1 group (5/12, 41.7%; *P* = .016). There were also other poor prognostic tendencies of advanced T stage, poor differentiation, perineural invasion, and large tumor size in high PSMD1 group, but without statistical significance (Table 1).

**Table 1:** Relationships between clinicopathological data and PSMD1 expression

Variables	n	Low PSMD1	High PSMD1	*P* value
**Gender**				.000
Male	**59**	48 (92.3%)	11 (91.7%)	
Female	**5**	4 (7.7%)	1 (8.3%)	
**Smoking**				.101
(-)	**26**	24 (46.2%)	2 (16.7%)	
(+)	**38**	28 (53.8%)	10 (83.3%)	
**Alcohol**				.778
(-)	**29**	24 (46.2%)	5 (41.7%)	
(+)	**35**	28 (53.8%)	7 (58.3%)	
**Age > 60**				.778
> 60	**29**	24 (46.2%)	5 (41.7%)	
< 60	**35**	28 (53.8%)	7 (58.3%)	
**Location**				.**002**
Tonsil	**46**	42 (80.8%)	4 (33.3%)	
Others	**18**	10 (19.2%)	8 (66.7%)	
**Pathologic T stage**				.351
T1-2	**55**	46 (88.5%)	9 (75.0%)	
T3-4	**9**	6 (11.5%)	3 (25.0%)	
**Pathologic N stage**				.810
N0-1	**30**	24 (46.2%)	6 (50.0%)	
N2-3	**34**	28 (53.8%)	6 (50.0%)	
**Overall stage**				.**005**
I-II	**52**	45 (86.5%)	6 (50.0%)	
III-IV	**12**	7 (13.5%)	6 (50.0%)	
**Differentiation**				.463
Class 1–2	**38**	32 (61.5%)	6 (50.0%)	
Class 3	**26**	20 (38.5%)	6 (50.0%)	
**Lymphovascular invasion**				.751
(-)	**39**	31 (59.6%)	8 (66.7%)	
(+)	**25**	21 (40.4%)	4 (33.3%)	
**Perineural invasion**				.252
(-)	**59**	49 (94.2%)	10 (83.3%)	
(+)	**5**	3 (5.8%)	2 (16.7%)	
**Extranodal extension**				.476
(-)	**21**	15 (51.7%)	6 (66.7%)	
(+)	**17**	14 (48.3%)	3 (33.3%)	
**Size**				.115
< 4 cm	**57**	48 (92.3%)	9 (75.0%)	
> 4 cm	**7**	4 (7.7%)	3 (25.0%)	
**Surgical margin**				.714
Free/close	**48**	38 (73.1%)	10 (83.3%)	
Involved	**16**	14 (26.9%)	2 (16.7%)	
**HPV**				.**016**
(-)	**15**	9 (17.3%)	6 (50.0%)	
(+)	**49**	43 (82.7%)	6 (50.0%)	
**Recurrence**				.314
(-)	**45**	38 (73.1%)	7 (58.3%)	
(+)	**19**	14 (26.9%)	5 (41.7%)	
**Surgical intent**				.196
Curative	**53**	45 (86.5%)	8 (66.7%)	
Salvage	**11**	7 (13.5%)	4 (33.3%)	
**Postoperative Radiotherapy**				.314
(-)	**19**	14 (26.9%)	5 (41.7%)	
(+)	**45**	38 (73.1%)	7 (58.3%)	

Fisher’s exact test and Pearson’s chi-square test were applied where appropriate. (HPV, Human papillomavirus)

### Survival analysis

**Fig. 4:**
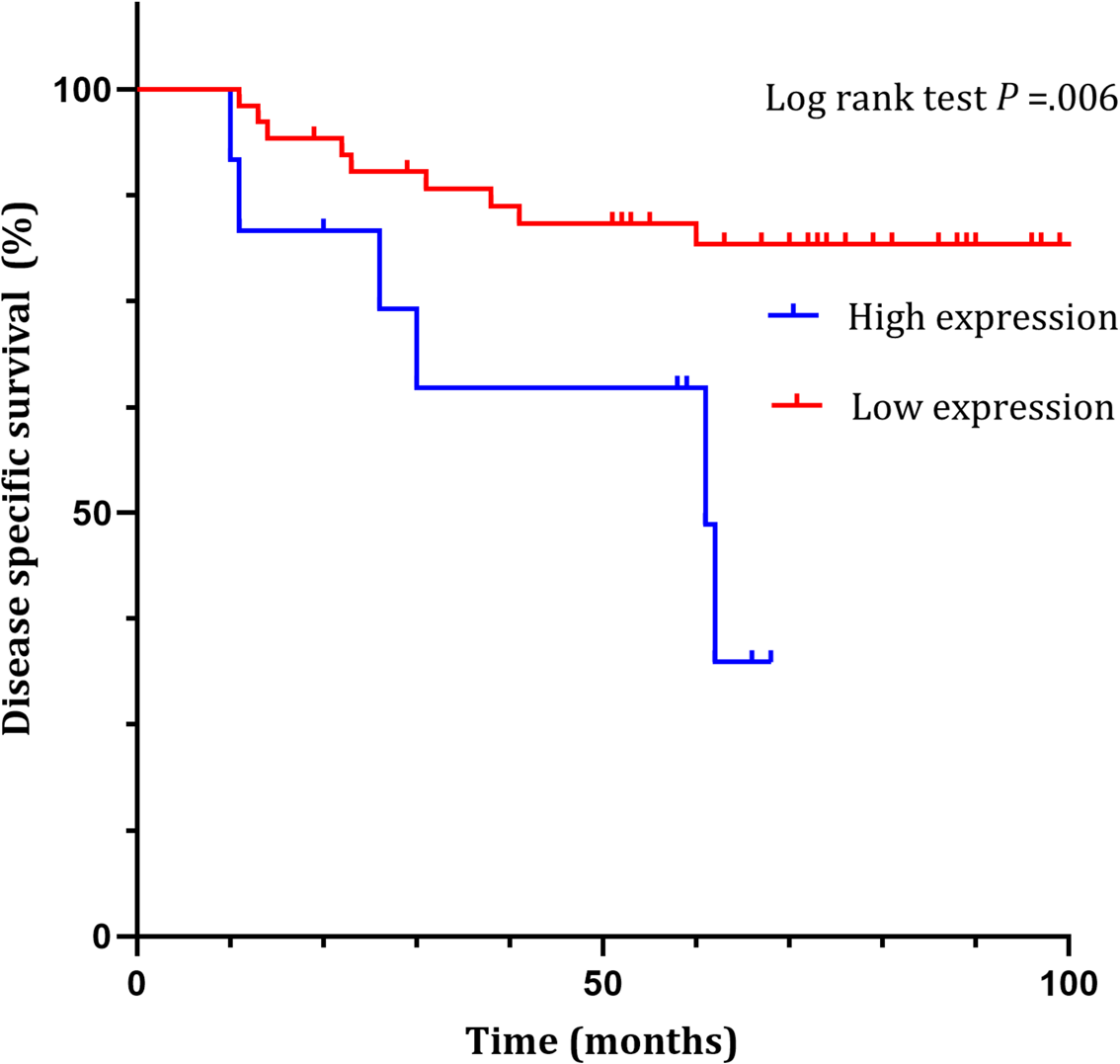
Disease specific survival graph analysis according to PSMD1 expression

Patients with PSMD1 high expression showed poorer DSS than in patients with PSMD1 low expression (*P* = .006 in log rank test) (Fig. 4).

Univariate analysis revealed that the PSMD1 expression, HPV positivity, pathologic T staging, and salvage operative intent affected DSS (*P* = .010, *P* = .002, *P* = .033, *P* = .002, respectively). In multivariate analysis, PSMD1 expression, pathologic T staging, and specimen age were found to be associated with DSS (*P* = .011, *P* = .025, *P* = .029, respectively). Odds ratio of PSMD1 expression was distinctive (5.924) (Table 2).

**Table 2:** Cox-regression analysis in univariate, multivariate clinicopathological data

Variables	Univariate *P* value	Multivariate *P* value	Hazard ratio (95% CI)
**PSMD1 expression**	**.010**	**.011**	5.924 (1.501–23.375)
**Age**	.517	.599	1.017 (0.954–1.085)
**Gender**	.780	.533	0.427 (0.029–6.183)
**Tumor location**	.456	.438	1.336 (0.420–13.869)
**HPV**	**.002**	.058	0.239 (0.054–1.052)
**Pathologic T stage**	**.033**	**.025**	2.606 (1.127–6.026)
**Pathologic N stage**	.938	.518	0.800 (0.407–1.574)
**Salvage surgery**	**.002**	.089	3.384 (0.831–13.783)
**Surgical margin**	.606	.323	2.413 (0.420–13.869)
**Postoperative radiotherapy**	.711	.827	1.201 (0.233–6.185)
**Specimen age**	.262	**.029**	0.738 (0.561–0.969)

‘Salvage surgery’ includes three cases of previous CCRT failure and one case of recurrence after previous surgery. (HPV, Human papillomavirus)

## Discussion

We investigated the prognostic role of PSMD1 expression in patients with OPSCC. PSMD1 was highly expressed in advanced pathologic staging, HPV negative tumor, and non-tonsillar cancer. Especially location was significant factor in PSMD1 nuclear expression. In multivariate analysis, PSMD1 revealed as an independent poor prognostic factor in OPSCC.

Although several studies have also investigated clinical significance of PSMD1 expression in head and neck cancer [12, 16], the survival analysis exhibited inconsistent results depending on subgroups. In a certain study, PSMD1 appeared even as a good prognostic factor in the survival analysis, which seems opposite from our results [16]. Our study ensured internal validity by specifically focusing on the OPSCC subgroup, accurately defining disease-specific causes of death, and conducting multivariate analysis that included the specimen age. Moreover, we ensured the significance of survival analysis by accurately defining “high expression” based on the H score and ROC curve analysis. There may be another impact from adoption of the 8th edition AJCC which published after 2016, restaging most HPV + OPSCCs to stage I or II [17].

There are various scientific explanations about PSMD1 as oncogene in literatures. PSMD1 gene encodes a subunit of the proteasome, 19S-RP, which regulates the degradation of various tumor suppressor and oncogenic proteins through the ubiquitin–proteasome pathway [18]. For instance, it has been reported that knocking down PSMD1 in a breast cancer cell line resulted in a decrease in the S phase and an increase in the G2/M phase, indicating inhibition of the cell cycle. Also, knockdown of PSMD1 silencing has been shown to activate the p53 pathway including p21 [8].

The role of p53 is already known to be associated with HPV infection in head and neck cancer. It has been observed that HPV-negative cases tend to have impaired p53 function particularly in the oropharynx [19]. P53 itself is typically wild type in HPV-positive cancers while the viral protein E6 directly induces the ubiquitination and degradation of p53 [20]. Indeed, if proteasome inhibitors such as bortezomib were used, proteasome is unable to degrade p53 even if E6 ubiquitinated it, thereby liberating p53 to resume its normal functions [21].

In addition to the cell cycle and the p53 pathway, various signaling pathways can be influenced by the proteasome. One example is the NF-kB pathway. NF-kB is a protein that promotes cell survival and contributes to cancer growth. IkB, on the other hand, is a protein that inhibits NF-kB. The proteasome plays a role in degrading IkB, thereby allowing NF-kB to remain active and promote cancer growth. Such explanation has been observed in multiple myeloma [22, 23]. These in vitro explanations support PMSD1 expression as a poor prognostic and oncogenic factor.

Our study may have inherent selection bias of IHC study towards early-stage cancers because of the requirement for surgical paraffin-embedded specimens. However, even in TCGA open data of head and neck cancer [12], where there is no selection bias for gene targets and subsites, PSMD1 was confirmed as a poor prognostic factor. This finding, along with various studies about PSMD1 as a poor prognostic factor in other types of cancer [5, 7–12], strengthens the external validity of our consistent study results. Also, survival rate of our study population decreased significantly in HPV negative group, with a *p*-value of less than 0.001 in the log-rank test (Supplementary Fig. 2). This valid tendency indicates that our study population is representative sample group of OPSCC. However, due to the lower prevalence of HPV-negative cases as known, we couldn't conduct a separate analysis for the HPV-negative group, which is a limitation of small sample size.

Because of the retrospective nature of the study, another limitation of the study is the potential confounding effect of other clinical features. We try to overcome lack of uniformity between the groups by including previous treatment history and staging on cox-regression multivariate analysis. Year of pathology is another main confounding concern. Staining tended to lighten with increasing pathology year, even in separate linear regression analysis (*P* < .001, Supplementary Fig. 3). To address this limitation, we incorporated specimen age when conducting Cox regression multivariate analysis. Nevertheless, significant *p*-values were obtained for variables that were consistent with the hypothesis.

## Conclusions

We confirmed that PSMD1 is an independent poor prognostic factor in squamous cell carcinoma of the oropharynx, and it is closely related to HPV negativity. Our results provide a foundation of further studies repurposing proteasome inhibitors for the treatment of OPSCC. Future in vitro studies should investigate the HPV-related downstream pathways and explore the effects of proteasome inhibitor alone or in combination with other anticancer agents.

### Supplementary Information

**Additional file 1: Supplementary Figure 1. **ROC curves of cellular, cytoplasmic, and nuclear H-score for the prediction of 5-year disease specific survival.

**Additional file 2: Supplementary Figure 2. **Kaplan-Meier survival curve demonstrating the relationship between disease specific survival (DSS) and the HPV positivity.

**Additional file 3: Supplementary Figure 3. **High PSMD1 expression as specimen age gets older.

**Additional file 4: Supplementary Table 1. **Clinicopathologic characteristics (HPV, Human papillomavirus).

## Data Availability

The data that support the findings of this study are available from the corresponding author upon reasonable request.

## Abbreviations

OPSCC: Oropharyngeal squamous cell carcinoma
HPV: Human papillomavirus
IHC: Immunohistochemistry
TMA: Tissue microarray
DSS: Disease-specific survival
OS: Overall survival
ROC: Receiver operating characteristic

## References

[1] Pryor DI, Solomon B, Porceddu SV (2011). The emerging era of personalized therapy in squamous cell carcinoma of the head and neck. Asia Pac J Clin Oncol.

[2] Porceddu SV, Scotte F, Aapro M, Salmio S, Castro A, Launay-Vacher V, Licitra L (2019). Treating patients with locally advanced squamous cell carcinoma of the head and neck unsuitable to receive cisplatin-based therapy. Front Oncol.

[3] Lecker SH, Goldberg AL, Mitch WE (2006). Protein degradation by the ubiquitin-proteasome pathway in normal and disease states. J Am Soc Nephrol.

[4] Weathington NM, Mallampalli RK (2014). Emerging therapies targeting the ubiquitin proteasome system in cancer. J Clin Invest.

[5] Muchtar E, Gertz MA, Magen H (2016). A practical review on carfilzomib in multiple myeloma. Eur J Haematol.

[6] Roeten MSF, Cloos J, Jansen G (2018). Positioning of proteasome inhibitors in therapy of solid malignancies. Cancer Chemother Pharmacol.

[7] Jonker PK, van Dam GM, Oosting SF, Kruijff S, Fehrmann RS (2017). Identification of novel therapeutic targets in anaplastic thyroid carcinoma using functional genomic mRNA-profiling: paving the way for new avenues?. Surgery.

[8] Okumura T, Ikeda K, Ujihira T, Okamoto K, Horie-Inoue K, Takeda S, Inoue S (2018). Proteasome 26S subunit PSMD1 regulates breast cancer cell growth through p53 protein degradation. J Biochem.

[9] Xiong W, Wang W, Huang H, Jiang Y, Guo W, Liu H, Yu J, Hu Y, Wan J, Li G (2019). Prognostic significance of PSMD1 expression in patients with gastric cancer. J Cancer.

[10] Boland K, Flanagan L, McCawley N, Pabari R, Kay EW, McNamara DA, Murray F, Byrne AT, Ramtoola Z, Concannon CG (2016). Targeting the 19S proteasomal subunit, Rpt4, for the treatment of colon cancer. Eur J Pharmacol.

[11] Bazzaro M, Lee MK, Zoso A, Stirling WL, Santillan A, Shih Ie M, Roden RB (2006). Ubiquitin-proteasome system stress sensitizes ovarian cancer to proteasome inhibitor-induced apoptosis. Cancer Res.

[12] Rubio AJ, Bencomo-Alvarez AE, Young JE, Velazquez VV, Lara JJ, Gonzalez MA, Eiring AM (2021). 26S proteasome Non-ATPase regulatory subunits 1 (PSMD1) and 3 (PSMD3) as putative targets for cancer prognosis and therapy. Cells.

[13] Doescher J, Veit JA, Hoffmann TK (2017). [The 8th edition of the AJCC cancer staging manual: updates in otorhinolaryngology, head and neck surgery]. HNO.

[14] Kwon S, Ahn SH, Jeong WJ, Jung YH, Bae YJ, Paik JH, Chung JH, Kim H (2020). Estrogen receptor α as a predictive biomarker for survival in human papillomavirus-positive oropharyngeal squamous cell carcinoma. J Transl Med.

[15] The Human Protein Atlas. https://www.proteinatlas.org/ENSG00000173692-PSMD1/pathology/head+and+neck+cancer. Accessed 18 Dec 2022.

[16] Lagadec C, Vlashi E, Bhuta S, Lai C, Mischel P, Werner M, Henke M, Pajonk F (2014). Tumor cells with low proteasome subunit expression predict overall survival in head and neck cancer patients. BMC Cancer.

[17] Machczynski P, Majchrzak E, Niewinski P, Marchlewska J, Golusinski W (2020). A review of the 8th edition of the AJCC staging system for oropharyngeal cancer according to HPV status. Eur Arch Otorhinolaryngol.

[18] Tsvetkov P, Adler J, Myers N, Biran A, Reuven N, Shaul Y (2018). Oncogenic addiction to high 26S proteasome level. Cell Death Dis.

[19] Maruyama H, Yasui T, Ishikawa-Fujiwara T, Morii E, Yamamoto Y, Yoshii T, Takenaka Y, Nakahara S, Todo T, Hongyo T (2014). Human papillomavirus and p53 mutations in head and neck squamous cell carcinoma among Japanese population. Cancer Sci.

[20] Kim TW, Choi SY, Ko YH, Baek CH, Son YI (2012). The prognostic role of p16 expression in tonsil cancer treated by either surgery or radiation. Clin Exp Otorhinolaryngol.

[21] Li C, Johnson DE (2013). Liberation of functional p53 by proteasome inhibition in human papilloma virus-positive head and neck squamous cell carcinoma cells promotes apoptosis and cell cycle arrest. Cell Cycle.

[22] Jang HH (2018). Regulation of protein degradation by proteasomes in cancer. J Cancer Prev.

[23] Liu L, Liu A, Dong J, Zuo Z, Liu X (2022). Proteasome 26S subunit, non-ATPase 1 (PSMD1) facilitated the progression of lung adenocarcinoma by the de-ubiquitination and stability of PTEN-induced kinase 1 (PINK1). Exp Cell Res.

