# Supplementary material for: PSMD1 as a prognostic marker and potential target in oropharyngeal cancer
Source: BMC Cancer. 2023 Dec 16;23:1242. doi: 10.1186/s12885-023-11689-2 (PMC10725586; doi:10.1186/s12885-023-11689-2)

**Supplementary Figure 1.** ROC curves of cellular, cytoplasmic, and nuclear H-score for the prediction of 5-year disease specific survival.


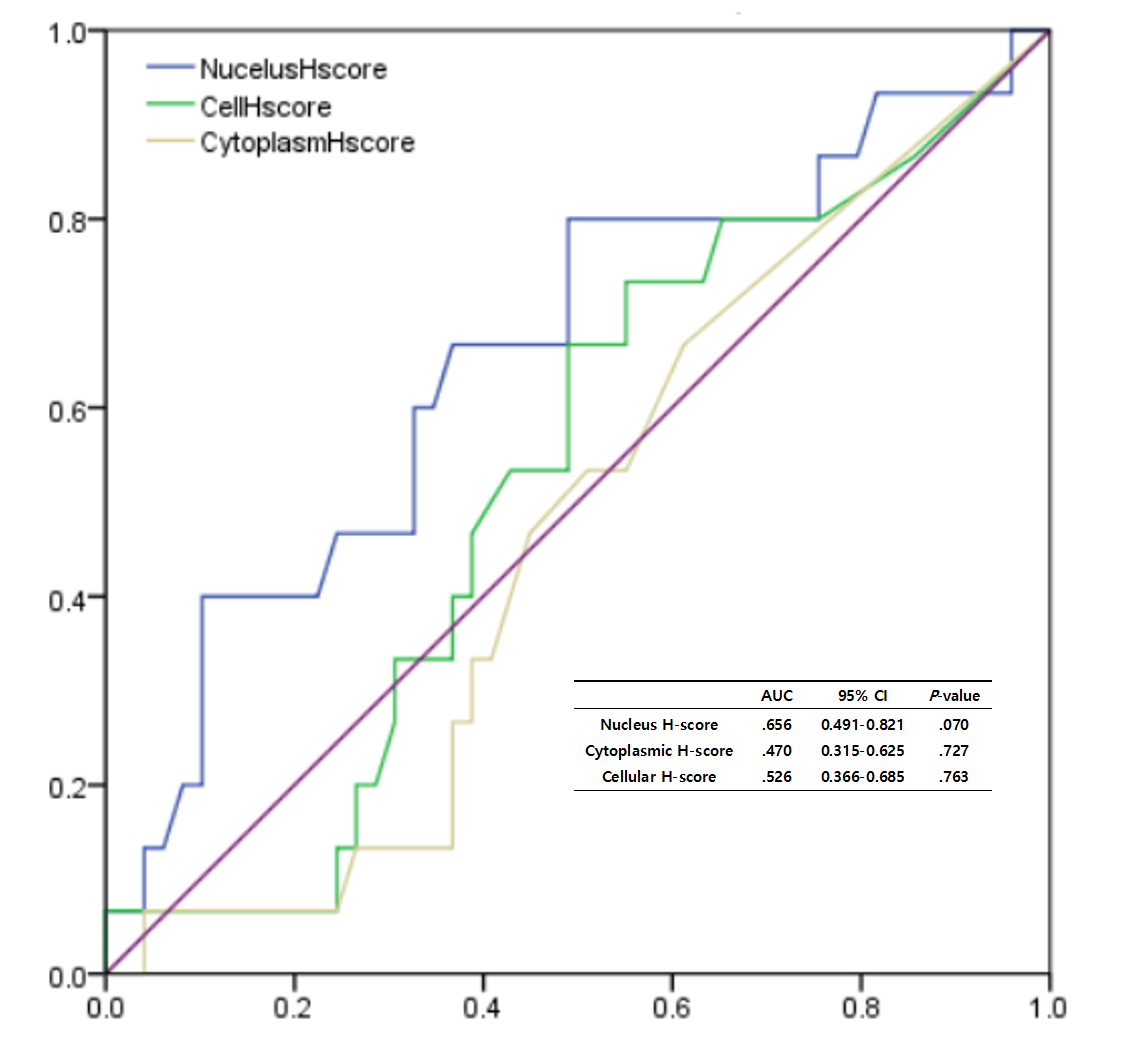

Supplement: Supplementary file 1 — Additional file 1: Supplementary Figure 1. ROC curves of cellular, cytoplasmic, and nuclear H-score for the prediction of 5-year disease specific survival. [file 12885_2023_11689_MOESM1_ESM.docx]
