# Supplementary material for: PSMD1 as a prognostic marker and potential target in oropharyngeal cancer
Source: BMC Cancer. 2023 Dec 16;23:1242. doi: 10.1186/s12885-023-11689-2 (PMC10725586; doi:10.1186/s12885-023-11689-2)

**Supplementary figure 2.** Kaplan-Meier survival curve demonstrating the relationship between disease specific survival (DSS) and the HPV positivity.


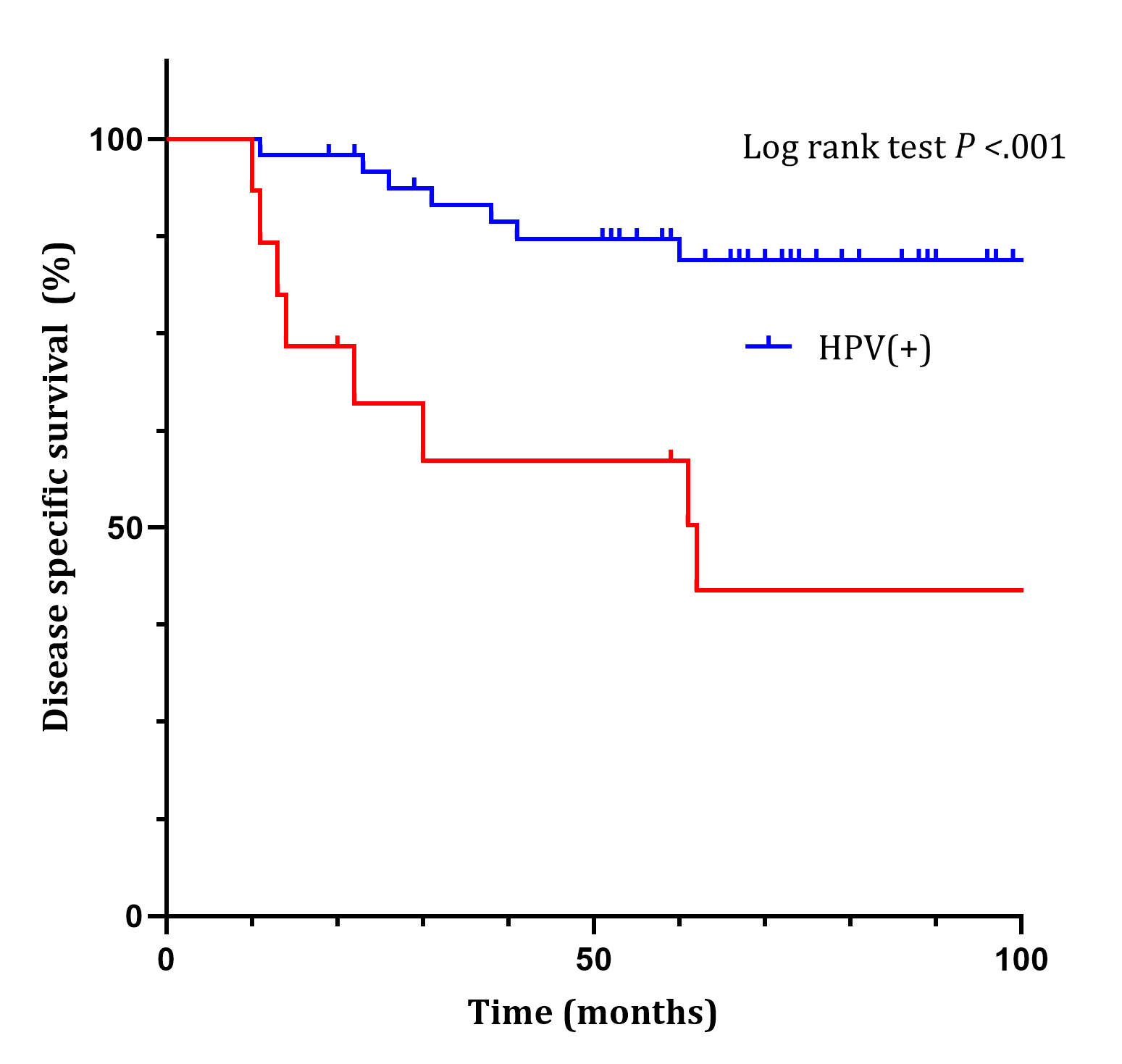

Supplement: Supplementary file 2 — Additional file 2: Supplementary Figure 2. Kaplan-Meier survival curve demonstrating the relationship between disease specific survival (DSS) and the HPV positivity. [file 12885_2023_11689_MOESM2_ESM.docx]
