# Supplementary figures and images for: PSMD1 as a prognostic marker and potential target in oropharyngeal cancer
Source: BMC Cancer. 2023 Dec 16;23:1242. doi: 10.1186/s12885-023-11689-2 (PMC10725586; doi:10.1186/s12885-023-11689-2)

**Supplementary figure 3.** High PSMD1 expression as specimen age gets older.


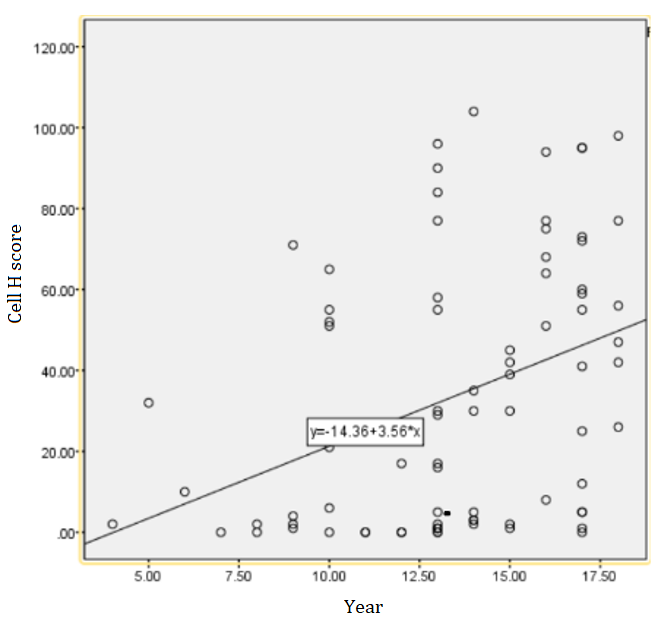

Supplement: Supplementary file 3 — Additional file 3: Supplementary Figure 3. High PSMD1 expression as specimen age gets older. [file 12885_2023_11689_MOESM3_ESM.docx]
