# Supplementary material for: PSMD1 as a prognostic marker and potential target in oropharyngeal cancer
Source: BMC Cancer. 2023 Dec 16;23:1242. doi: 10.1186/s12885-023-11689-2 (PMC10725586; doi:10.1186/s12885-023-11689-2)
Supplement: Supplementary file 4 — Additional file 4: Supplementary Table 1. Clinicopathologic characteristics (HPV, Human papillomavirus). [file 12885_2023_11689_MOESM4_ESM.docx]

**Supplementary table 1. Clinicopathologic characteristics** (HPV, Human papillomavirus)

| **Sex** | **n** | % |
| --- | --- | --- |
| male | 59 | 92.2 |
| female | 5 | 7.8 |
| **Age** | **n** | % |
| <60y | 35 | 54.7 |
| >=60y | 29 | 45.3 |
| **Location** | **n** | % |
| tonsil | 46 | 71.9 |
| base of tongue | 8 | 12.5 |
| soft palate | 4 | 6.3 |
| pharyngeal wall | 5 | 7.8 |
| uvula | 1 | 1.6 |
| **HPV related** | **n** | % |
| HPV- | 15 | 23.4 |
| HPV+ | 49 | 76.6 |
| **T staging** | **n** | % |
| T1 | 15 | 23.4 |
| T2 | 40 | 62.5 |
| T3-4 | 9 | 14.1 |
| **N staging** | **n** | % |
| Nx | 10 | 15.6 |
| N0 | 15 | 23.4 |
| N1 | 22 | 34.4 |
| N2-3 | 17 | 26.6 |
| **Overall staging** | **n** | % |
| I | 38 | 59.4 |
| II | 13 | 20.3 |
| III | 5 | 7.8 |
| IV | 8 | 12.5 |
| **Differentiation** | **n** | % |
| Well | 8 | 12.5 |
| moderate | 30 | 46.9 |
| poor | 26 | 40.6 |
| **Surgical intent** | **n** | % |
| Curative surgery | 53 | 82.8 |
| Limited biopsy | 11 | 17.2 |
